# Transitions in the wintertime near‐surface temperature inversion at Dome C, Antarctica

**DOI:** 10.1002/qj.3450

**Published:** 2019-03-08

**Authors:** Peter Baas, Bas J. H. van de Wiel, Erik van Meijgaard, Etienne Vignon, Christophe Genthon, Steven J. A. van der Linden, Stephan R. de Roode

**Affiliations:** ^1^ Geoscience & Remote Sensing Delft University of Technology Delft the Netherlands; ^2^ Weather & Climate Models Royal Netherlands Meteorological Institute De Bilt the Netherlands; ^3^ Environmental Remote Sensing Laboratory École Polytechnique Fédérale de Lausanne Lausanne Switzerland; ^4^ Institute for Geosciences and Environmental Research Université Grenoble Alpes/CNRS/IRD Grenoble France; ^5^ Sorbonne Université, École Normale Supérieure, PSL Research University, École Polytechnique, CNRS, Laboratoire de Météorologie Dynamique, LMD/IPSL Paris France

**Keywords:** Antarctic atmosphere, observational data analysis, regime transition, single‐column model, stable boundary layer

## Abstract

In this work we study the dynamics of the surface‐based temperature inversion over the Antarctic Plateau during the polar winter. Using 6 years of observations from the French–Italian Antarctic station Concordia at Dome C, we investigate sudden regime transitions in the strength of the near‐surface temperature inversion. Here we define “near‐surface” as being within the domain of the 45‐m measuring tower. In particular, we consider the strongly nonlinear relation between the 10‐m inversion strength (*T*_10m_ – *T*_s_) and the 10‐m wind speed.

To this end, all individual events for which the 10‐m inversion strength increases or decreases continuously by more than 15 K in time are considered. Composite time series and vertical profiles of wind and temperature reveal specific characteristics of the transition from weak to very strong inversions and vice versa. In contrast to midlatitudes, the largest variations in temperature are not found at the surface but at a height of 10 m.

A similar analysis was performed on results from an atmospheric single‐column model (SCM). Overall, the SCM results reproduce the observed characteristics of the transitions in the near‐surface inversion remarkably well. Using model output, the underlying mechanisms of the regime transitions are identified. The nonlinear relation between inversion strength and wind speed at a given level is explained by variations in the geostrophic wind speed, changes in the depth of the turbulent layer and the vertical divergence of turbulent fluxes. Moreover, the transitions between different boundary layer regimes cannot be explained without considering the contribution of subsidence heating.

## 1 INTRODUCTION

One of the most prominent climatological features of the East Antarctic Plateau is the strong surface‐based temperature inversion. This is especially true for the austral winter, as during the summer period the inversion is eroded by the diurnal cycle of solar radiation. During winter, the inversion strength generally exceeds 20 K and may at times approach 40 K (Dalrymple, 1966; Phillpot and Zillman, 1970; Connolley, 1996; Hudson and Brandt, 2005; Genthon *et al.,* 2013; Pietroni *et al.,* 2014; Vignon *et al.,* 2017b).

The surface‐based inversion develops due to radiative cooling of the surface. The radiative loss is balanced by heat transport towards the surface through the ice/snow pack and from the atmospheric boundary layer (e.g., Schlatter, 1972; Cerni and Parish, 1984; van de Berg *et al.,* 2007). The air adjacent to the surface cools through vertical divergence of the sensible heat flux and the radiative flux. This atmospheric cooling is compensated for by heat advection resulting from the large‐scale dynamics. In the lowest part of the troposphere, the main source of energy on the dome‐shaped Antarctic Plateau is provided by subsiding air motions that are driven by the divergent flow pattern of the katabatic drainage flows surrounding the plateau (e.g., Businger and Rao, 1968; Parish and Bromwich, 1987; King and Turner, 1997; van de Berg *et al.,* 2008; Vignon *et al.,* 2018).

Recently, Vignon *et al*. (2017b) showed the existence of two contrasting regimes in the 10‐m temperature inversion (*T*_10m_ – *T*_s_) at Dome C, which is located on the high central ridge of the East Antarctic Plateau at 3,233 m above sea level. The first regime is characterized by relatively strong winds and a weak inversion in the order of 5 K. In the second regime, the winds are weak but the temperature difference over the lowest 10 m may be as large as 25 K. The transition between these two states is abrupt and occurs at a 10‐m wind speed of 5–7 m/s. These findings corroborate the conceptual model that was proposed by van de Wiel *et al*. (2017). The relation between the inversion strength and the wind speed was previously studied by Hudson and Brandt (2005). They found a clear relation between the inversion strength between 22 and 2 m and the wind speed at 10 m above ground level at the Amundsen–Scott South Pole Station. The relevance of the ambient wind speed in determining the height and intensity of the inversion was already acknowledged by Dalrymple (1966).

While previous studies have provided valuable insights into the climatology of the surface‐based inversion and the characteristics of the two contrasting regimes, a systematic study of the transitions between the two regimes is lacking. In this paper, we bridge this knowledge gap by presenting a detailed analysis of the time evolution of the transitions between strong (calm wind) and weak (strong wind) inversions. To this end, all individual events for which the 10‐m inversion strength increases or decreases significantly over time were selected from 6 years of observations obtained from a 45‐m meteorological tower at the French–Italian Concordia Station at Dome C. The selected events are divided into two subsets: those that include transitions from a weak to a very strong inversion and those for the reversed path. For both subsets, composite time series and vertical profiles of relevant variables are constructed. We will show that this approach provides new insights into the characteristics of regime transitions in the near‐surface inversion strength. To emphasize the generic validity of our results, transitions in the 40‐m inversion strength (*T*_40m_ – *T*_s_) will also be discussed. In addition to the observations, results from a numerical model will be used to disentangle the underlying mechanisms of the regime transitions.

We only consider data from the extended austral winter, hereafter defined as the period from April 1 to September 30. In this period the sun is below the horizon most of the time. In the first weeks of April and in the second half of September in particular the solar radiation during the daytime may still be significant. Nevertheless, these months are included in our analysis as the temporal variations in the inversion strength are more reminiscent of the large and irregular regime transitions characteristic of the polar winter than of the diurnal cycles observed during summer.

In this work, we aim to contribute to a better understanding of regime transitions in the near‐surface temperature inversion over the Antarctic Plateau by studying selected events in which the inversion strength changes significantly. Here, we define “near‐surface” as being within the domain of the 45‐m tower. Specifically, we aim to:

- understand the dynamics between the near‐surface wind and the inversion strength by analysing the time evolution of regime transitions selected from 6 years of observations;
- assess the ability of a numerical model to reproduce the observed behaviour;
- identify the dominating processes that drive the transitions in the inversion strength by analysing momentum and temperature budgets from model output.

This paper is structured as follows. Section 2 describes the observations and the model. It also provides a brief overview of the boundary layer climatology at Dome C. The methodology is explained in Section 3, while Section 4 presents the results. After analysing variations in the mean profiles for the selected transition cases, the underlying mechanisms of the regime transitions are investigated by considering the momentum and temperature budgets as diagnosed from model output. The results are further discussed in Section 5. Section 6 summarizes the conclusions.

## 2 IN SITU OBSERVATIONS, DOME C BOUNDARY LAYER CLIMATOLOGY AND MODEL DESCRIPTION

### 2.1 Site description and in situ observations

The French–Italian Concordia Station at Dome C is located at a distance of approximately 900 km from the coast (75°06′ S, 123°20′ E, 3,233 m above sea level). The terrain consists of a homogeneous snow desert with no discernible slope (less than 1‰). Since 2009, the lower atmospheric boundary layer has been monitored continuously at Dome C (Genthon *et al.,* 2010; 2013). The data collected since then form a unique natural laboratory in which to study the dynamics of stably stratified boundary layers. In this work, we use 6 years of observations obtained between 2011 and 2016.

The flatness and homogeneity of Dome C prevent the local generation of katabatic winds as noted by Aristidi *et al*. (2005) and King *et al*. (2006). They showed that, as a result, observed wind speeds at Dome C are relatively small compared to other Antarctic stations (cf. Sanz Rodrigo *et al.,* 2013). This is confirmed by model studies such as, for example, Parish and Bromwich (1987), and van den Broeke and van Lipzig (2003).

Most of the time, a southerly large‐scale flow advects cold and dry air towards Dome C. As a consequence, the sky is often cloud‐free, the total water content is low (Ricaud *et al.,* 2015) and the annual snow accumulation amounts to only 8 cm/year (i.e., the equivalent of less than 3 cm/year liquid water; Genthon *et al.,* 2016). However, strong low‐pressure systems over the austral ocean occasionally bring relatively warm and moist maritime air towards the East Antarctic Plateau. These so‐called “warming events” may give rise to a sudden temperature increase of tens of degrees over several hours (Argentini *et al.,* 2001; Gallée and Gorodetskaya, 2010). They modify the surface radiation budget (Ricaud *et al.,* 2017) and are likely responsible for most of the snowfall at Dome C (Genthon *et al.,* 2016). As shown by Pietroni *et al*. (2014) and Vignon *et al*. (2018), these events may lead to a sudden erosion of the near‐surface temperature inversion at Dome C.

At Dome C, wind, temperature and moisture are continuously measured at six levels along a 45‐m high tower with the instruments facing the dominant wind direction (Genthon *et al.,* 2013). The tower is located 1 km to the west of the main buildings of Concordia Station. In 2012, an auxiliary 2.5‐m mast was set up approximately 400 m from the main tower to sample the lowest metres above the surface in more detail. Measuring heights are summarized in Table 1. Note that due to the snow accumulation of 8 cm per year, the height of the mast and the effective measurement heights decrease over the years. As the present study predominantly uses observations from the 10‐m level, this effect is ignored in the remainder of this work.

**Table 1:** Data availability of wind and temperature observations from April to September for the years 2011–2016

T	Surface	0.4 m	0.8 m	2.9 m	10.3 m	17.6 m	25.0 m	32.4 m	41.6 m
	*92%*	*48%*	*48%*	*100%*	*100%*	*100%*	*100%*	*100%*	*100%*
U	—	1.3 m	2.3 m	3.3 m	8.9 m	18.2 m	25.5 m	32.9 m	41.3 m
		*25%*	*40%*	*82%*	*82%*	*82%*	*77%*	*59%*	*74%*

Indicated instrument heights are those measured in January 2015. Underscores indicate observations from the auxiliary 2.5‐m mast that was set up in 2012. The aerovane at 1.3 m was only installed in January 2015.

Wind measurements are performed with Young 05103/6 propeller aerovanes. The manufacturer‐stated measurement accuracy is 0.3 m/s with a starting threshold of 1 m/s. Wind speed observations for wind directions between 50° and 100° are discarded because of flow obstruction by the tower. Temperature measurements are performed with Vaisala HMP155 thermo‐hygrometers in aspirated radiation shields with a measurement range of −80 to 60°C. At −50°C the manufacturer‐stated accuracy is ±0.35°C. Data availability for the extended winter periods from 2011 to 2016 is indicated in Table 1. For our analysis we use half‐hourly means. More details on the measurement site and the instrumentation can be found in Vignon (2017).

Radiation measurements are obtained from the Baseline Surface Radiation Network (BSRN, Lanconelli *et al.,* 2011). The surface skin temperature is determined from the surface radiative fluxes using the Stefan–Boltzmann law for a grey body (snow emissivity taken as 0.99; Vignon, 2017).

Obviously, the harsh conditions at the Antarctic Plateau present a severe challenge for performing measurements, especially during winter. Hitherto, it seemed impossible to perform any sort of continuous high‐frequency measurements with sonic thermo‐anemometers. Several brief measurement campaigns have been carried out during the summer period (Vignon *et al.,* 2017a), but as we restrict ourselves to the polar winter, we do not discuss them.

Daily 1200 UTC (2000 h local time) radio soundings were obtained from the Italian National Program of Research in Antarctica (PNRA) Project “Routine Meteorological Observations at Station Concordia”. The data are freely available at http://www.climantartide.it.

### 2.2 Monthly climatology of the Dome C boundary layer

**Figure 1:**
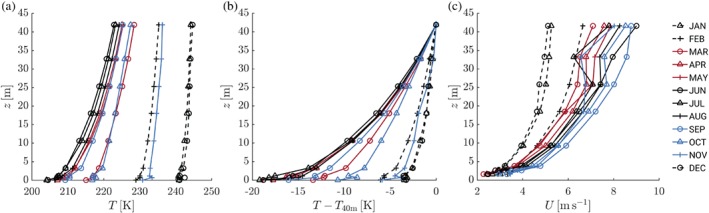
Monthly averaged profiles of temperature (a), temperature relative to *T*_40m_ (b) and wind speed (c) [Colour figure can be viewed at wileyonlinelibrary.com].

For illustration purposes, Figure 1 shows average temperature and wind speed profiles for each month of the year. During austral summer, the average temperature is about 240 K. Despite the sun being continuously above the horizon, the average temperature profile is still stably stratified; the average inversion strength along the tower is approximately 5 K. The wind speed is relatively weak and well mixed in comparison with the other seasons.

In the winter months, the near‐surface temperatures are more than 30 K lower than in summer. The temperature difference between the top of the mast and the surface is 18 K on average. During the extended austral winter, the surface‐based inversion evolves into a quasi‐stationary state: the monthly averaged near‐surface temperature and the inversion strength remain relatively constant from April to September (e.g., Tomasi *et al.,* 2011; Pietroni *et al.,* 2014). The absence of a well‐defined minimum in the wintertime temperature gave rise to the frequently used term “coreless winter” (Wexler, 1958, and earlier references therein).

In the winter half‐year, wind speeds are clearly higher than in summer. Apart from synoptic effects, this may be a result of increased stability of the atmospheric boundary layer, such that turbulent friction on the large‐scale flow is reduced. The wind speed is at maximum in the spring months from September to November. The relatively low values at 32 m during the winter period are caused by instrument malfunction.

### 2.3 Model

To analyse the dynamics of the transitions in the near‐surface inversion, we use model output from the single‐column model (SCM) of the Regional Atmospheric Climate Model (RACMO), which is based on the physics module of Cy31r1 of the European Centre for Medium‐Range Weather Forecasts (ECMWF)'s Integrated Forecasting System (IFS; ECMWF, 2007). The main difference with the IFS model is the parameterization of vertical mixing. Instead of the original first‐order closure model, the SCM utilizes a turbulent kinetic energy (TKE) closure model. The individual terms of the TKE equation are parameterized in terms of the local mean gradients of wind, temperature and TKE. For the computation of the mixing efficiencies of momentum and heat, the diagnostic length‐scale formulation proposed by Lenderink and Holtslag (2004) is used. The settings of the turbulence scheme are similar to those of Baas *et al*. (2018). Using the same model, they adequately simulated the stable boundary layer at Cabauw, the Netherlands, for a wide range of stability conditions. Radiation transport is modelled with the Rapid Radiative Transfer Model (RRTM) scheme. Interactions with the surface and soil dynamics are represented by the Tiled ECMWF Scheme for Surface Exchanges over Land (TESSEL), which consists of four layers in the soil, a “skin layer” with zero heat capacity, a vegetation layer and a single snow layer. For more details on the model we refer the reader to ECMWF (2007).

Inspired by the fourth GEWEX (Global Energy and Water Exchanges) Atmospheric Boundary Layer Studies (GABLS) intercomparison study (Bazile *et al.,* 2015), in which a summertime diurnal cycle at Dome C is simulated, the depth of the snow layer is given a fixed value of 5 cm. With this value the heat capacity of the snow layer is such that it responds realistically to changing forcing conditions on the time‐scale of 1 day. The role of the underlying soil layers is negligible, especially during the polar winter. In the present SCM simulations no vegetation is prescribed. The roughness length for momentum is set to 10^−4^ m, and the roughness length for heat is set to 5 × 10^−5^ m. Although the observed estimates of the roughness length show considerable spread, these values seem realistic for the inland Antarctic Plateau (van den Broeke *et al.,* 2005; Dutra *et al.,* 2015; Vignon *et al.,* 2017a).

The SCM is run for the period 2011–2016, which is similar to the period of the observational dataset. The forcing data consist of initial profiles and hourly time–height fields of the geostrophic wind, vertical velocity and advective tendencies of wind, temperature and humidity. These were obtained from a three‐dimensional (3D) climate simulation with the RACMO21P model that was performed as a contribution to the Coordinated Regional Climate Downscaling Experiment (CORDEX; Giorgi *et al.,* 2009) dedicated to Antarctica. The model domain of this simulation encompassed the Antarctic continent, the horizontal resolution was 50 km and 40 vertical model levels were employed. At the ocean and the lateral boundaries, the model was forced every 6 hr by ERA‐Interim (Dee *et al.,* 2011). The atmospheric and land–ice states in the domain interior were allowed to evolve freely, while the initial model state was taken from the ERA‐Interim reanalysis state verified on January 1, 1979 at 0000 UTC.

RACMO21P combines the dynamical core of the High Resolution Limited Area Model (HiRLAM; Undén *et al.,* 2002) with the physics package of the ECMWF's IFS, including the default highly diffusive mixing scheme. Compared to the reference model version RACMO2.1 (van Meijgaard *et al.,* 2008), RACMO21P includes a series of model updates specifically implemented to increase performance and realism in polar regions, in particular a multilayer snow scheme (Ettema *et al.,* 2010), an improved formulation of snow albedo (Kuipers Munneke *et al.,* 2011) and a parametric scheme to represent snow drift processes (Lenaerts *et al.,* 2012).

The SCM simulations are initialized at the start of each month. The model state is nudged to the 3D model fields with a time‐scale of 24 hr. This prevents the SCM from drifting away from the 3D model fields and avoids the formation of spurious inertial oscillations near the surface. At the same time, the time‐scale of 24 hr is long enough to give the fast boundary layer physics enough freedom to establish their own unique state (Neggers *et al.,* 2012). The SCM grid consists of 90 vertical levels. Near the surface the grid spacing is roughly 6 m, with the lowest level located approximately 3 m above the surface. This high‐resolution grid configuration is adopted from the GABLS4 intercomparison study (E. Bazile, personal communication). Model values at specific heights, for example 10 and 40 m as used in the present study, are obtained by linear interpolation from model levels.

As a proof of principle, Appendix A presents wintertime averaged vertical profiles of temperature, wind speed, specific humidity, net long‐wave radiation, vertical velocity and horizontal temperature advection. Throughout the troposphere, ERA‐Interim, RACMO21P and the SCM are closely correlated. The models represent the observed temperature and wind speed profiles accurately. Near the surface, the SCM captures observed vertical gradients in wind and temperature better than ERA‐Interim and RACMO21P as a result of the more realistic vertical mixing parameterization.

## 3 METHODOLOGY

**Figure 2:**
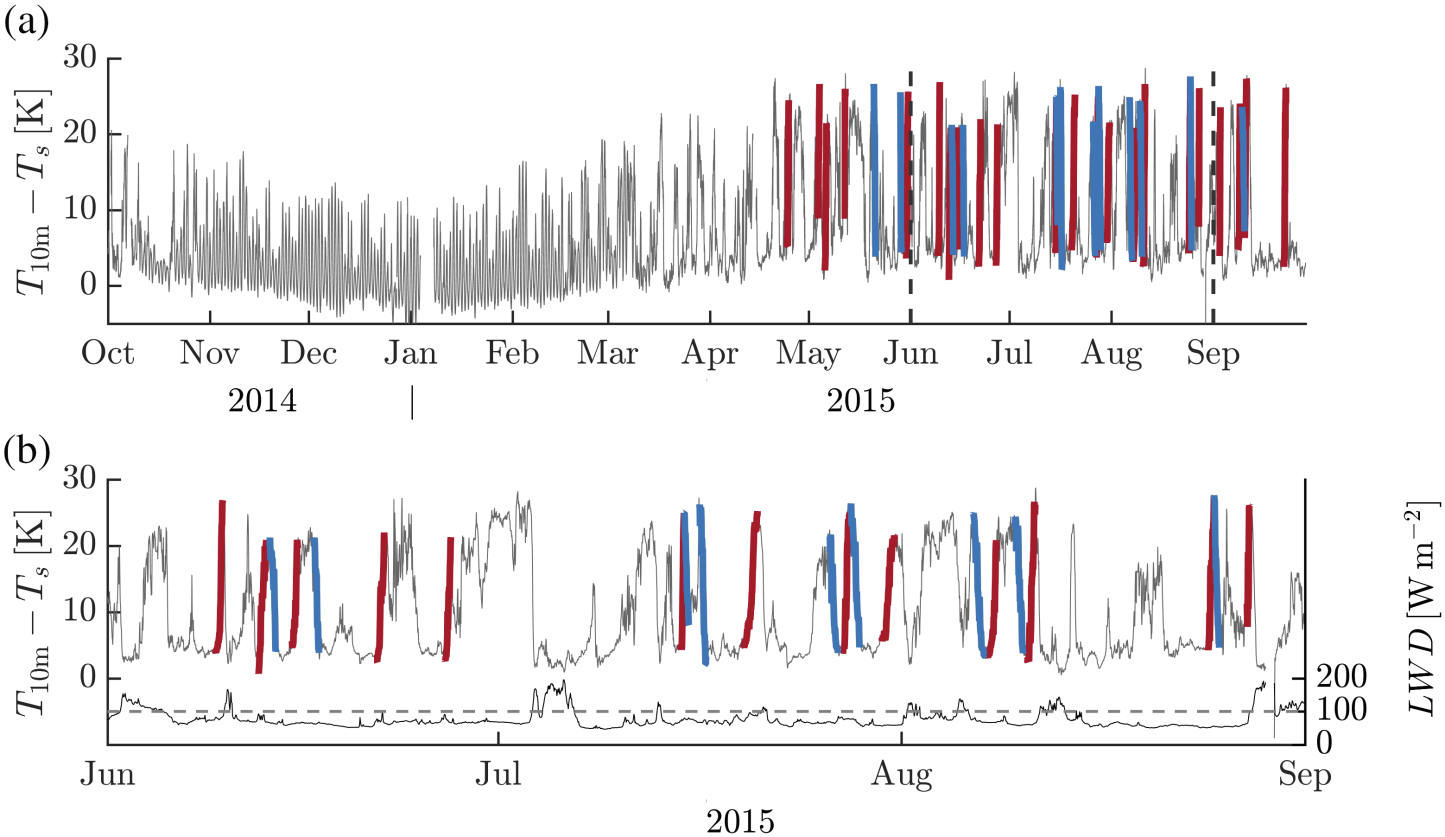
One‐year time series of the temperature difference between 10 m and the surface (a). Red (blue) segments indicate selected events with formation (erosion) of the 10‐m inversion. Data for three winter months (June, July and August) are replotted in (b) together with the time series of *LW*_*d*_. The dashed line indicates the 100 W/m^2^ threshold value on *LW*_*d*_ [Colour figure can be viewed at wileyonlinelibrary.com].

To study the regime transitions of the boundary layer at Dome C, we take the time series of the inversion strength as a starting point. Following Vignon *et al*. (2017b), we consider the temperature difference between 10 m and the surface. To minimize the impact of rapid fluctuations, a moving average of 3 hr is applied. For illustration purposes, Figure 2a presents a 1‐year period of this time series. During summer, a clear diurnal cycle is present with stably stratified “nights” and convective “days.” Around March, the amplitude of the diurnal cycle diminishes quickly. During winter, large fluctuations in the inversion strength remain with values ranging from about 5 to 25 K. Figure 2b provides a more detailed picture of the polar winter period by showing only data from the months of June, July and August, 2015. In order to investigate whether a preferred time‐scale exists for the winter transitions, we performed a spectral analysis of 10‐m inversion strength. Unlike the daily cycle in summer, no specific dominant time‐scale was found (not shown).

Earlier studies mainly related different variables of a dataset without considering the temporal relation between data points (e.g., with the use of scatter plots). In contrast, here we study selected individual events by preserving the temporal relation between the considered variables (e.g., temperature and wind). From the 6‐year dataset, all extended‐winter events have been selected for which the *T*_*10m*_ − *T*_*s*_ temperature difference increased or decreased continuously in time with more than 15 K. As we are primarily interested in boundary layer dynamics, we exclude cases with overcast conditions. To this end, the incoming long‐wave radiation *LW*_*d*_ is required to be less than 100 W/m^2^. Petenko *et al*. (2014) suggested 80 W/m^2^ as an upper threshold value for cloud‐free conditions. We prefer a somewhat higher value, as this leads to a considerable increase in the number of selected cases. A similar threshold value was used by Vignon *et al*. (2018).

In total, 76 (63) transition cases of inversion formation (erosion) were found that also satisfied the criterion on *LW*_*d*_. The results of the present work are mainly based on an analysis of these two subsets of selected cases: by constructing composite time series and vertical profiles we derive generic features of the regime transitions. As indicated in Table 2, the number of detected transition events that satisfy the imposed criteria varies considerably from year to year.

**Table 2:** Number of observed transition cases per year

Year	2011	2012	2013	2014	2015	2016	Total
Inversion formation	6	13	13	15	22	7	76
Inversion erosion	8	7	16	14	12	6	63

In Figure 2, selected events are highlighted by red and blue line segments. A similar analysis has been applied to the SCM results. In this case, 107 (111) events were found in which the modelled 10‐m inversion strength increased (decreased) continuously by more than 15 K.

In Figure 2b, *LW*_*d*_ observations are also included. Clearly, the threshold value imposed on *LW*_*d*_ excludes quite a few cases of significant change of the inversion strength. Many of these events are associated with synoptic events that advect warm and moist air from northerly directions. A notable example, discussed in detail by Vignon *et al*. (2018), occurs on July 5. All transition events that *do* satisfy the imposed criterion on *LW*_*d*_ are characterized by southerly flow. We conclude that the applied criterion on *LW*_*d*_ effectively circumvents the selection of transition events related to synoptic warming events (see also Appendix B).

Obviously, applying different thresholds on the required change in inversion strength leads to a different case selection. For example, when the selection threshold is lowered from 15 to 10 K, the number of inversion formation (erosion) events increases to 150 (136). Moreover, while taking *T*_10m_ – *T*_s_ as a reference may be consistent with the analysis of Vignon *et al*. (2017b), using the *T*_40m_ – *T*_s_ temperature difference would be an equally valid approach. Setting the threshold to 15 K as before, this results in 74 (87) events of continuous formation (erosion) of the inversion. These events will be analysed as well. In Section 4.4 it will be shown that their dynamics is qualitatively similar to that of the *T*_10m_ – *T*_s_ events.

## 4 RESULTS

In this section, the characteristics of transitions in the wintertime inversion strength are presented. All results are based on the 6‐year dataset described in Section 2.1 or the model output comprising a similar time period. First, we discuss the general distribution of the inversion strength for different heights above the surface (Section 4.1). An analysis of the selected individual events follows in Section 4.2. Section 4.3 focuses on the underlying mechanisms by analysing temperature and momentum budgets as diagnosed from model output. Transitions in the 40‐m inversion (*T*_40m_ – *T*_s_) are discussed in Section 4.4.

### 4.1 Distribution of the wintertime inversion strength

**Figure 3:**
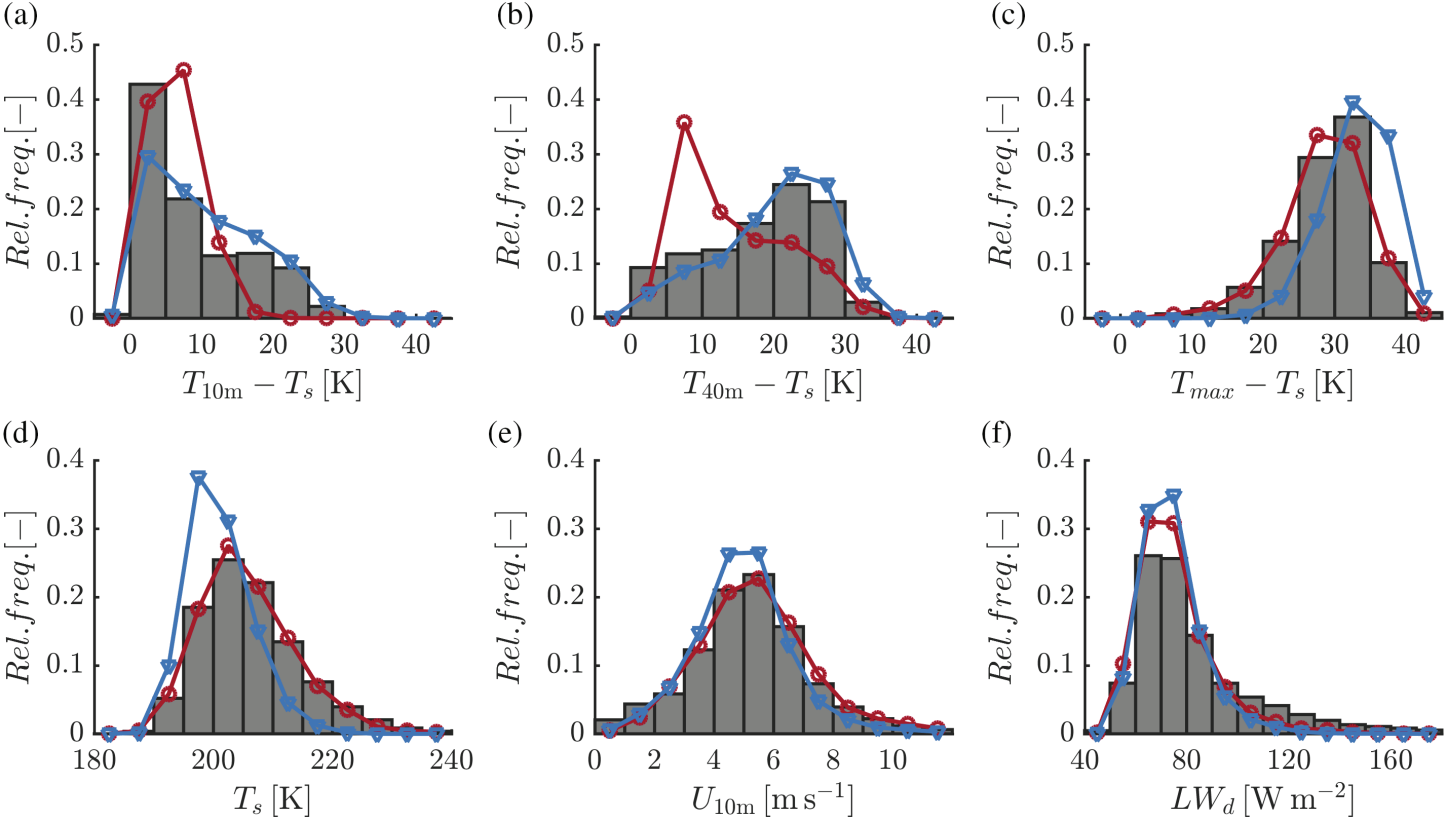
Wintertime frequency distributions of (a) the 10‐m inversion strength, (b) the 40‐m inversion strength, (c) the total surface‐based inversion, (d) the surface temperature, (e) the 10‐m wind speed, and (f) the downward long‐wave radiation. The histograms represent the observations, the red lines (circles) the 3D driver model and the blue lines (triangles) the single‐column model (SCM) [Colour figure can be viewed at wileyonlinelibrary.com].

Figure 3 presents the frequency distribution of the inversion strength for various heights above the surface as derived from the 6‐year dataset. The distribution of the 10‐m inversion is positively skewed, with most occurrences between 0 and 5 K (Figure 3a). At the same time, inversion strengths up to 25 K occur a significant portion of the time. Interestingly, the distribution of the total surface‐based inversion (the maximum tropospheric temperature obtained from daily radio sounding minus the surface temperature) is strongly negatively skewed (Figure 3c). The total inversion strength exceeds 30 K for almost 50% of the time. The strength of the 40‐m inversion is also skewed negatively, but with a more uniform distribution than the other two cases (Figure 3b).

The SCM (blue lines, triangles) reproduces the different frequency distributions surprisingly accurately. The 3D driver model (red lines, circles) is strongly biased towards weaker inversion strengths in the lowest 40 m above ground level. Clearly, in contrast to the TKE scheme of the SCM, the first‐order scheme with the “enhanced‐mixing” stability functions of RACMO21P is not able to reproduce the very strong temperature gradients near the surface. At the same time, its representation of the total surface‐based inversion is accurate. Also, the 3D model represents the distributions of *T*_s_ and *U*_10m_ very well (Figure 3d,e). The SCM underestimates *T*_s_, which may be related to the simple one‐layer snow scheme that is used. The models reproduce the positively skewed distribution of *LW*_*d*_, although values over 100 W/m^2^ are underrepresented. In the remainder of this work only results from the SCM will be presented.

### 4.2 Analysis of selected events: mean variables

**Figure 4:**
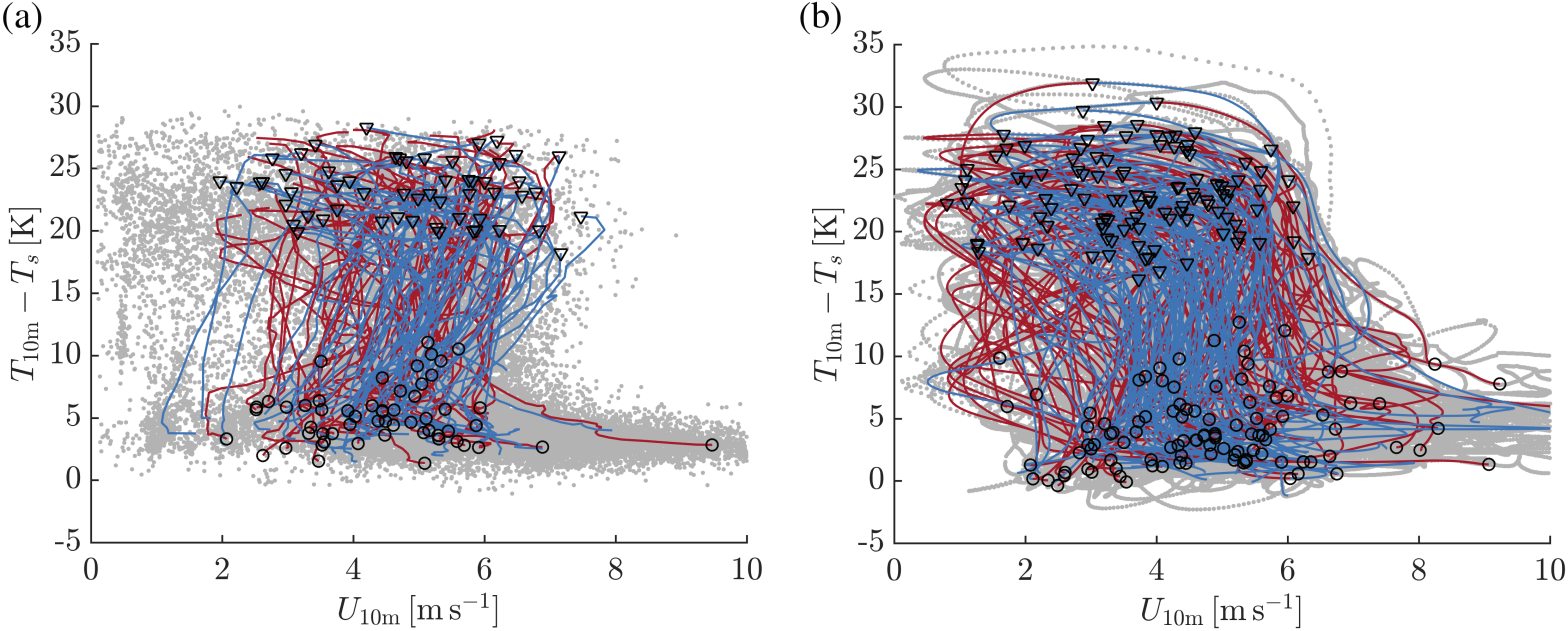
Observed (a) and modelled (b) relation between the 10‐m temperature inversion and wind speed. Red (blue) lines indicate time series of selected events of significant inversion formation (erosion). The starting point of each inversion formation (erosion) event is indicated with a circle (triangle). Grey dots indicate all wintertime data (*LW*_*d*_ < 100 W/m^2^) [Colour figure can be viewed at wileyonlinelibrary.com].

Figure 4 presents the relation between the 10‐m inversion strength and the 10‐m wind speed. The red (blue) lines represent time series of inversion formation (erosion) for all selected individual events with a continuous change in the inversion strength of more than 15 K. The grey symbols represent the other data. The selected events are representative of the overall relation between inversion strength and wind speed.

As argued by Vignon *et al*. (2017b), the observations (Figure 4a) clearly show the existence of two regimes. Wind speeds lower than ∼ 4 m/s are associated with very strong 10‐m inversions of 20–25 K. In contrast, wind speeds higher than ∼7 m/s correspond to relatively weak inversions of order 5 K. As emphasized by the time series of the individual events, the transition between the two regimes is highly nonlinear in the chosen phase space. In fact, the relation between the inversion strength and the wind speed reflects the shape of an inverted “S,” as was anticipated by van de Wiel *et al*. (2017).

As shown in Figure 4b, the SCM reproduces the observed relation between inversion strength and wind speed reasonably well. The extremes of the inversion strength are captured accurately. The nonlinearity in the transition is reproduced, even though the degree of “back folding” appears less pronounced than in the observations.

**Figure 5:**
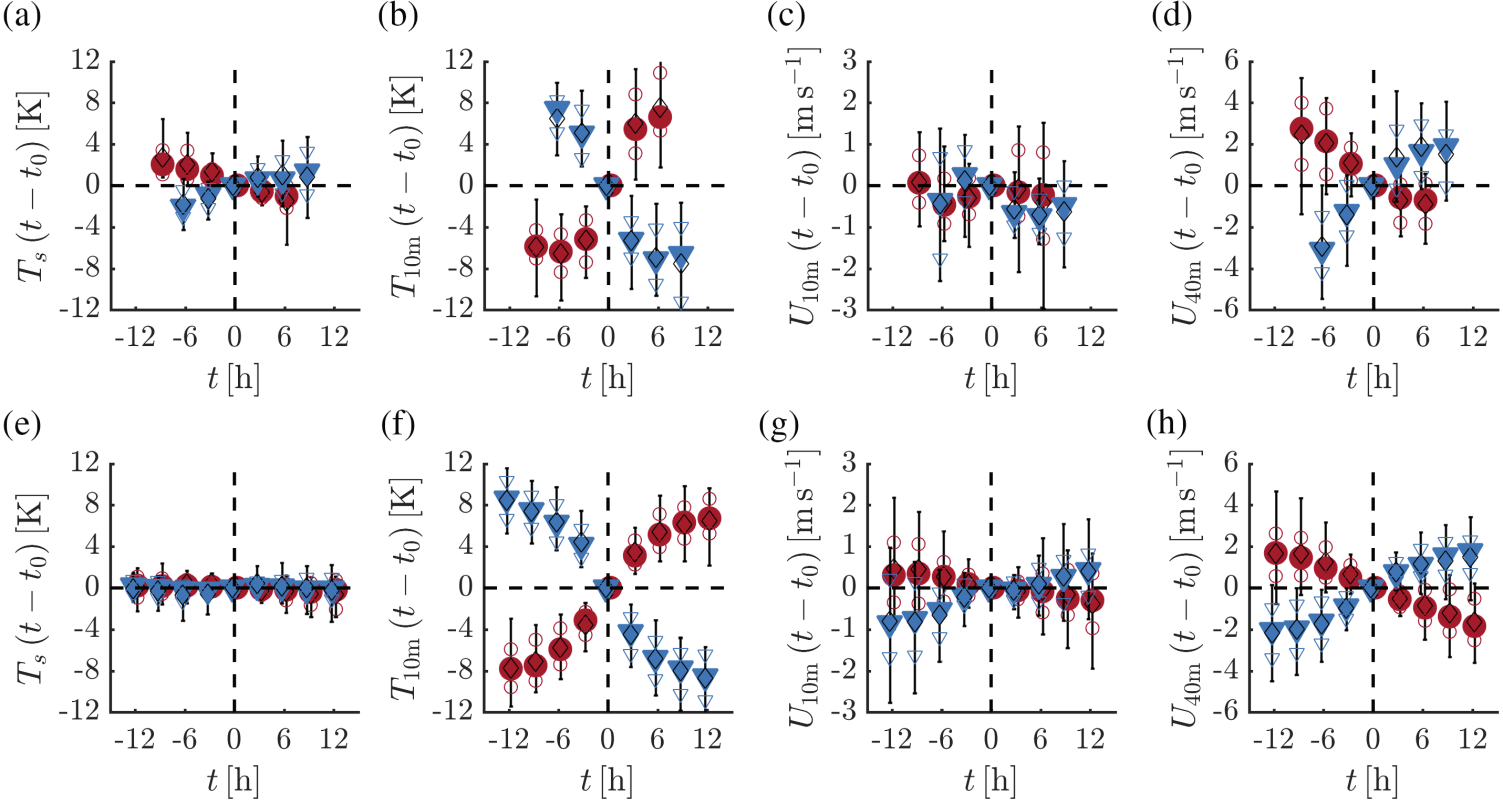
Observed (top panels) and modelled (bottom panels) composite time series of the change in *T*_s_ (a,e), *T*_10m_ (b,f), *U*_10m_ (c,g) and *U*_40m_ (d,h) for selected events with inversion formation (red circles) and erosion (blue triangles). Filled symbols indicate median values, open symbols the 25th and 75th percentiles, and bars the 10th and 90th percentiles. Open black diamonds indicate average values. Only data points for which more than 25% of the selected events are available are indicated [Colour figure can be viewed at wileyonlinelibrary.com].

The selected events are further analysed in Figure 5, which presents composite time series of *T*_s_, *T*_10m_, *U*_10m_ and *U*_40m_ for both the observations (top panels) and the model results (bottom panels). For each event, *t* is set to 0 hr at the moment in time that the inversion strength equals the average value of the start and end times. The value at *t* = 0 hr is subtracted from the time series. As a result, by definition all time series are 0 at *t* = 0 hr. As the duration of the selected transition events varies, the number of events decreases for larger (absolute) values of *t*. Figure 5 includes only data for time steps for which more than 25% of the selected events are present.

Figure 5a,b shows that transitions in the inversion strength are predominantly associated with large variations in *T*_10m_. In contrast, the changes in the surface temperature are relatively small. This is markedly different from the conditions at midlatitudes, where fluctuations in the near‐surface inversion strength are generally the result of a diurnal cycle that is driven from the surface. The two transition types show symmetric behaviour when considering the evolution of *T*_10m_ and *T*_s_: during the transition the absolute rate of change of the inversion strength is equal for both cases.

Figure 5c shows the relative course of the 10‐m wind speed. For the events in which the inversion weakens significantly (blue triangles), an initial increase in the median of the 10‐m wind speed is followed by a clear decrease, after which it strengthens again. The section of weakening 10‐m winds corresponds to the “folding back” feature in Figure 4. In contrast, the wind speed at 40 m increases continuously (Figure 5d) with changes in the order of 5–7 m/s. For events with a significant increase of the inversion strength, opposite reasoning can be applied.

Figure 5e–h presents composite time series based on model output. Roughly, the evolution of *T*_s_, *T*_10m_, *U*_10m_ and *U*_40m_ corresponds to the observed equivalents. The much larger variation in *T*_10m_ compared to the surface temperature is well represented. Although the increase in *U*_10m_ is delayed around *t* = 0 hr, a temporary decrease is lacking. This corresponds to the underestimation of the “back folding” in Figure 4b.

Table 3 presents the distribution of the length of the observed transition events. The median value of the duration of the transitions is close to 14 hr for both the formation and erosion of the 10‐m inversion. The difference in the distribution of both transition types is small. This is also true for the modelled transitions. However, with a median value of 27 hr the transition events selected from the model output take much longer than the ones selected from the observations. Yet, this difference in duration may be misleading, as Figure 5 also shows that in the model the bulk of the regime transition essentially occurs within 12 hr.

**Table 3:** Distribution of the time length of observed transition cases

Percentile	10th (hr)	25th (hr)	50th (hr)	75th (hr)	90th (hr)
Formation (*n* = 76)	7.6	10.8	13.8	17.0	19.0
Erosion (*n* = 63)	9.0	12.1	14.0	16.4	19.6

**Figure 6:**
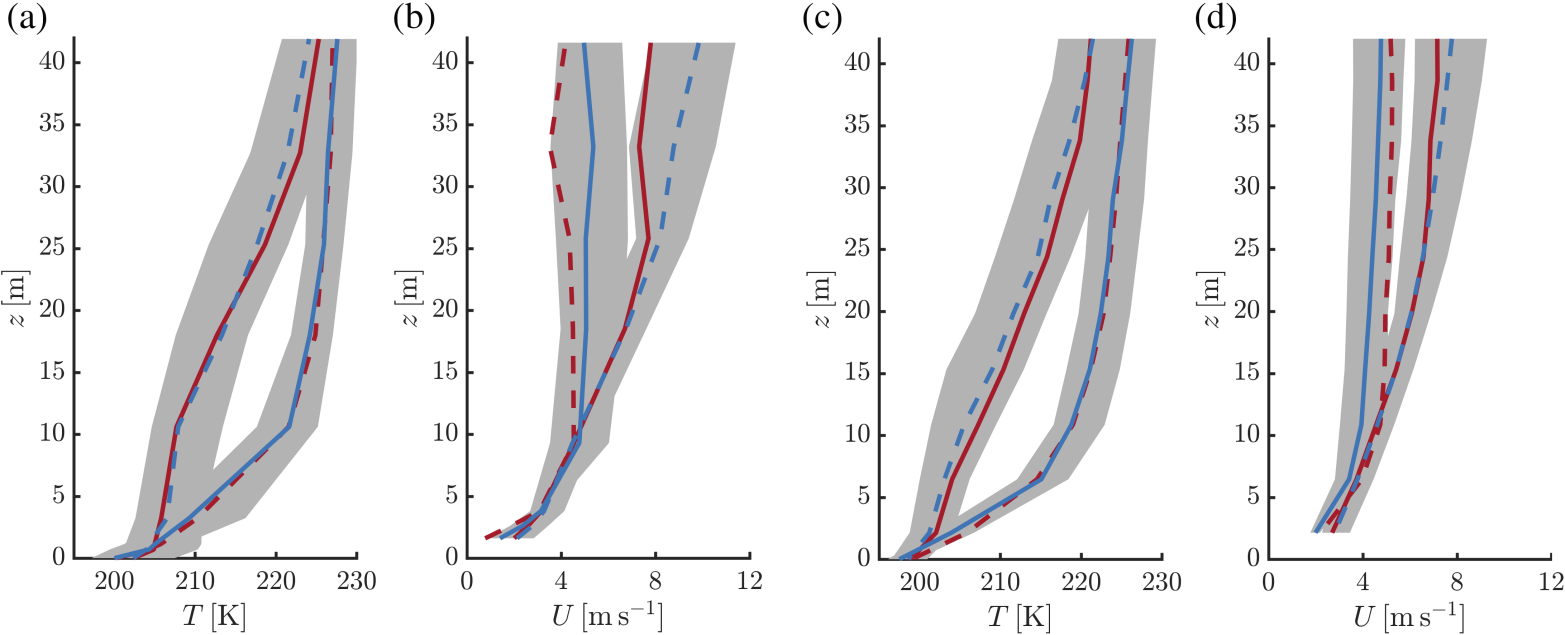
Observed (a,b) and modelled (c,d) composite profiles (median values) of temperature (a,c) and wind speed (b,d) for the selected events of formation (red) and erosion (blue) of the 10‐m inversion. Solid lines represent *t* = −6 hr, dashed lines *t* = 6 hr. For the case of inversion erosion the shaded areas indicate the spread between the 25th and 75th percentiles [Colour figure can be viewed at wileyonlinelibrary.com].

Figure 6 presents observed (a,b) and modelled (c,d) composite profiles of temperature and wind speed at *t* = −6 and *t* = 6 hr. Roughly, these time steps correspond to the start and end times of the regime transitions. In the very stable state, the temperature profile shows a clear exponential (convex) shape. In this case, the wind speed is almost constant above the 10‐m level. In the weakly stable state the temperature profile has a “convex–concave–convex” shape with two inflection points at approximately 7 and 20 m. The wind speed increases continuously with height along the measurement tower. The contrast between the two states is emphasized by the fact that for a significant part of the domain the 25th and 75th percentiles of the temperature profiles do not overlap. The model‐based composite profiles closely resemble those based on observations.

The difference in shape between temperature profiles in weakly stable and very stable conditions is a well‐known feature of the stable boundary layer. Genthon *et al*. (2013) discussed the changing curvature in temperature profiles at Dome C. For the same location, Vignon *et al*. (2017b) discussed two typical examples and related their occurrence to distinct stable boundary layer regimes. Van Ulden and Holtslag (1985) presented cases with contrasting curvature using data from Cabauw. Using a one‐dimensional model, Garratt and Brost (1981) argued that the concave part of the temperature profile is dominated by turbulent cooling, while radiative processes dominate the convex part of the profile.

### 4.3 Analysis of selected events: momentum and temperature budgets

We analysed the evolution of temperature and wind speed for more than 100 events in which the 10‐m inversion strength changes rapidly by more than 15 K. These strong fluctuations in the near‐surface inversion with time mark the transition between a weakly stable and a very stable state. During the transition the 10‐m inversion strength and 10‐m wind speed are closely related. A natural follow‐up step would be an analysis of the temperature and momentum budgets in order to obtain greater insight into the underlying dynamics of the system. However, an assessment of turbulent fluxes cannot be done based on observational data, as the extremely harsh conditions of the Antarctic winter prevent reliable turbulence measurements. Instead, we utilize the good correspondence between the observations and the SCM results: the SCM realistically reproduces observed features of the transition. This suggests that studying the dynamics of this phenomenon on the basis of model output is justified.

**Figure 7:**
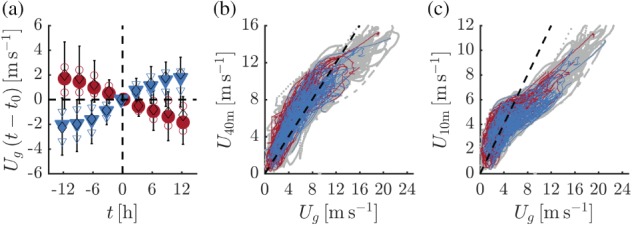
Composite time series of the change in *U*_*g*_ for selected events (a). Symbols are as for Figure 5. Relation between the wind speed at 40 m (b) and 10 m (c) and *U*_*g*_ (the dashed line indicates the 1:1 line). Events with inversion formation (erosion) are indicated in red (blue). Grey dots indicate all wintertime data (*LW*_*d*_ < 100 W/m^2^) [Colour figure can be viewed at wileyonlinelibrary.com].

First, we analyse the external forcing of the transitions in the 10‐m inversion strength. Figure 7a shows composite time series of the modelled geostrophic wind speed *U*_*g*_ for the selected events (*U*_*g*_ is evaluated at a height of 10 m). Periods with formation (erosion) of the 10‐m inversion correspond to periods with a continuously decreasing (increasing) *U*_*g*_. This suggests that the fluctuations in the near‐surface inversion strength are directly driven by changes in the horizontal pressure gradient. Figure 7b,c examines the relation between *U*_*g*_ and the 40‐m and 10‐m wind speeds, respectively. Apparently, for geostrophic wind speeds less than 10 m/s, the 40‐m wind speed is on average equal to *U*_*g*_. However, for stronger geostrophic forcings the wind speed at 40 m lags behind. In general, this suggests that during the polar winter at Dome C the 40‐m wind speed can be used as a proxy for an external forcing of the boundary layer, at least for wind speeds up to 10 m/s (see also the correspondence between Figures 7a and 5h). Qualitatively, the relation between the 10‐m wind and *U*_*g*_ (Figure 7c) resembles the one between the 40‐m wind and *U*_*g*_. However, the 10‐m wind can already be seen to lag behind *U*_*g*_ for values larger than around 5 m/s. Comparison with Figure 4b indicates that this value is close to the maximum wind speed for which very strong (>20 K) 10‐m inversions occur.

**Figure 8:**
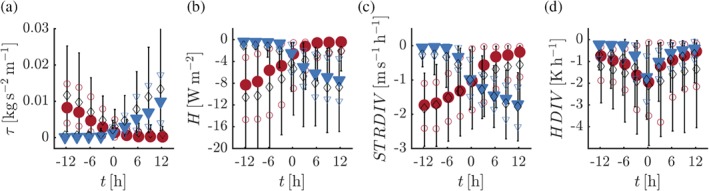
Composite time series of τ (a) and *H* (b) at 10 m for selected events. The wind and temperature tendencies as a result of vertical divergence of τ (*STRDIV*) and *H* (*HDIV*) at 10 m are given in (c) and (d), respectively. Colours and symbols are as for Figure 5 [Colour figure can be viewed at wileyonlinelibrary.com].

To gain better insight into the large temperature fluctuations at 10‐m height, we consider composite time series of the turbulent stress *τ* and the sensible heat flux *H* at this level (Figure 8a and Figure 8b, respectively). Also shown are the wind and temperature tendencies at this level as a result of the vertical divergence of *τ* (Figure 8c) and *H* (Figure 8d), which are indicated by *STRDIV* and *HDIV*, respectively. Clearly, the transitions in the 10‐m inversion strength are related to large fluctuations in the turbulent quantities. When the 10‐m inversion is weak, the turbulent fluxes at 10 m are significant (Figure 8a,b). In the case of strong inversions, turbulence is virtually absent at this level.

As shown in Figure 7a, erosion of the 10‐m inversion (blue triangles) is associated with a marked increase of *U*_*g*_. Figure 6 shows that, as a result, the wind speed in the lowest 40 m tends to increase. However, changes in the 10‐m wind speed are small (Figure 5c,g). In fact, at around *t* = 0 hr a temporary drop occurs in *U*_10m_ (especially in the observations). Figure 8c suggests that the temporary drop in *U*_10m_ coincides with a sudden increase of the 10‐m stress divergence. Thus, while the increase in the pressure gradient (or *U*_*g*_) tends to accelerate the wind speed, at 10 m this increase is counteracted by the effect of turbulent friction. At the moment when stress divergence starts retarding the 10‐m wind speed, vigorous heat flux divergence causes a significant decrease of the 10‐m temperature (Figure 8d). This leads to the erosion of the 10‐m inversion (Figure 5b,f).

The case of inversion formation essentially mirrors that of inversion erosion. Now the increase of the inversion strength concurs with a decrease of turbulent fluxes at 10 m. The decrease of *U*_*g*_ (Figure 7a) is partly compensated for by a strong reduction in the stress divergence. Again, this leads to a strongly nonlinear relation between the 10‐m inversion strength and *U*_10m_.

**Figure 9:**
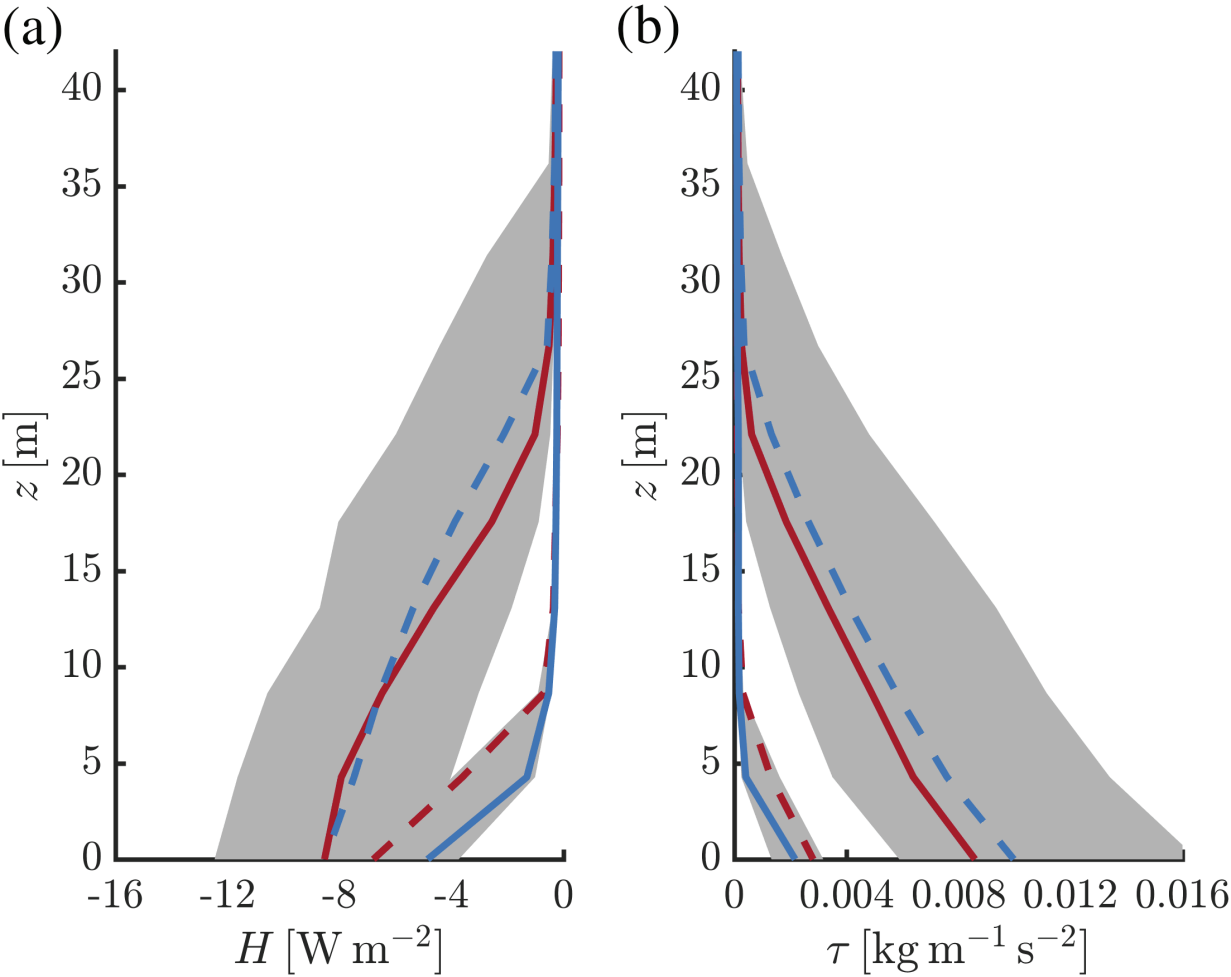
Composite vertical profiles of *H* (a) and *τ* (b) for selected events of inversion formation (red) and erosion (blue). Solid lines represent *t* = −6 hr, dashed lines *t* = 6 hr. For the case of inversion erosion the shaded areas indicate the spread between the 25th and 75th percentiles [Colour figure can be viewed at wileyonlinelibrary.com].

Figure 9 presents composite (median) profiles of *H* and *τ* at *t* = −6 and 6 hr. Strong inversions are associated with a very shallow turbulent layer of only 5–10 m. Weak inversions are characterized by much deeper turbulent layers with depths of several tens of meters. The variability between cases is large, as shown by the shaded areas that indicate the 25th and 75th percentiles.

The present analysis suggests that the strengthening and weakening of the 10‐m inversion, as well as the associated variations in the 10‐m wind speed, are closely related to the depth of the turbulent (boundary) layer being thinner or thicker than 10 m. This is consistent with the mean wind and temperature profiles shown in Figure 6, which indicated that both observed and modelled profiles are indicative of a turbulent boundary layer of significant depth in case of a weak 10‐m inversion and of a boundary layer dominated by radiative processes in case of very strong inversions.

**Figure 10:**
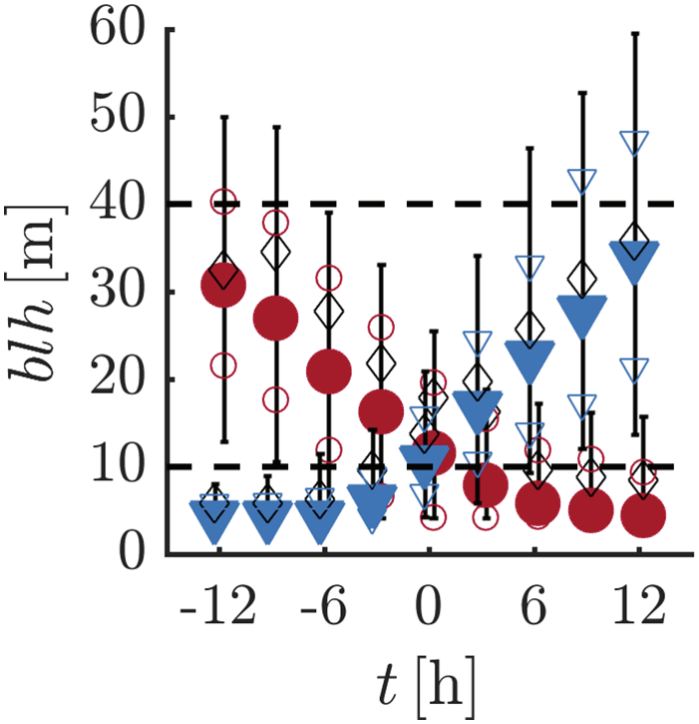
Composite time series of the boundary layer depth. Colours and symbols are as for Figure 5 [Colour figure can be viewed at wileyonlinelibrary.com].

Figure 10 presents the evolution of the turbulent boundary layer depth (defined as the height where the stress has been reduced to 5% of its surface value) for the selected cases. Strong 10‐m inversions are related to boundary layers of 10 m and less, while in the weakly stable state the depth of the turbulent layer regularly exceeds 30–40 m. Similar results are found by Petenko *et al*. (2019) who analysed SODAR observations obtained during the polar winter at Dome C.

So far, many features of the dynamics of the inverted “S” curve at Dome C could be explained by analysing the evolution of the external forcings and the (vertical divergence of the) turbulent fluxes. However, one essential element remains elusive, namely the increase in the 10‐m temperature (cf. Figure 5b,f) associated with formation of the 10‐m inversion. While heat flux divergence may explain the decrease of *T*_10m_ in the case of inversion erosion, it certainly does not contribute to its increase in the case of inversion formation (Figure 8d).

**Figure 11:**
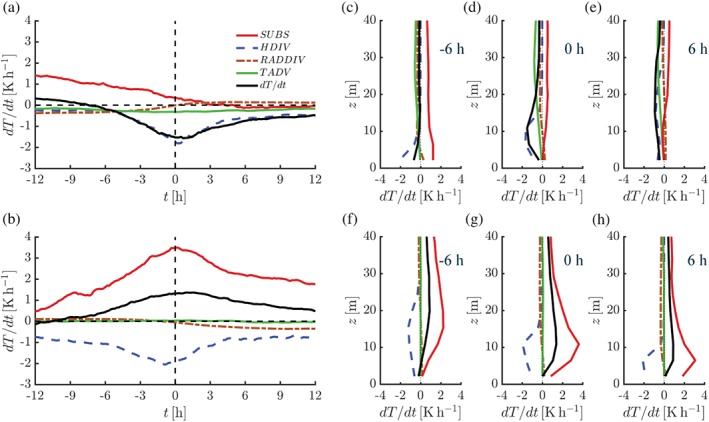
Composite time series of the terms of the temperature budget equation at *z* = 10 m for selected events with 10‐m inversion erosion (a) and formation (b). *HDIV* refers to the heat flux divergence, *RADDIV* to the radiation divergence, *TADV* to the horizontal temperature advection and *SUBS* to the subsidence heating. Vertical profiles at *t* = −6, 0 and 6 hr are shown in (c–e) for inversion erosion and in (f–h) for inversion formation. Median values are presented [Colour figure can be viewed at wileyonlinelibrary.com].

As a next step, we inspect the individual terms of the temperature budget equation at 10‐m height to identify the dominating processes. The temperature budget equation is given as

$$\begin{array}{cc} \frac{\partial T}{\partial t} & =\underset{\text{Horizontal advection}}{\underbrace{-\left(u\frac{\partial T}{\partial x}+v\frac{\partial T}{\partial y}\right)}}\underset{\text{Subsidence}}{\underbrace{-w\left(\frac{\partial T}{\partial z}+\frac{g}{c_{p}}\right)}}\\ & \;\underset{\text{Radiation}}{\underbrace{-\frac{1}{\rho c_{p}}-\frac{\partial Q_{*}}{\partial z}}}\underset{\text{Turbulence}}{\underbrace{-\frac{1}{\rho c_{p}}-\frac{\partial \mathrm{THF}}{\partial z}}}, \end{array}$$

where (*u*, *v*, *w*) indicate the components of the wind vector, $Q_{*}$ is the net radiation, *c*_*p*_ is the isobaric heat capacity per unit mass of dry air, *ρ* is the air density, *g* is the magnitude of gravity and *THF* is the turbulent heat flux. Figure 11a shows composite time series of the various terms of the temperature budget, evaluated at 10 m above the surface, for the case of significant erosion of the 10‐m inversion. In this case, the average 10‐m temperature tendency peaks at almost −2 K/hr (cf. Figure 5f). The rapid cooling at 10 m above the surface around *t* = 0 hr is mainly associated with increased (modulus) values of the turbulent heat flux divergence. Also, the gradual decrease of subsidence heating during the transition period contributes to a lowering of the 10‐m temperature. The horizontal advection of temperature is small compared to the other terms in the heat budget. The initially small radiative cooling in the case of a strong inversion gradually evolves into a small radiative warming when the inversion has become weak. The vertical profiles indicate the strong heat flux divergence at *t* = 0 hr. The depth of the turbulent layer, as reflected in the profiles of the turbulent heat flux divergence, increases with time.

As heat flux (and radiation) divergence generally lead to cooling, the substantial warming at 10‐m height during formation of the inversion (Figures 5 and 6) must be a result of heat advection through the large‐scale dynamics. The model results indicate that the increase in 10‐m temperature associated with a strengthening of the 10‐m inversion is caused by increased warming due to subsidence (Figure 11b). The importance of subsidence heating in the boundary layer of the Antarctic Plateau was stressed in earlier model studies by van de Berg *et al*. (2007) and Vignon *et al*. (2018). The contribution of horizontal advection is negligible. The effect of radiative flux divergence is small. Consistent with earlier results, the vertical profiles indicate a substantial decrease in the depth of the turbulent layer (Figure 11f–h).

The maximum warming rate of almost 4 K/hr in the case of formation of the 10‐m inversion may seem surprisingly high. However, a combination of a vertical velocity of −1 mm/s and a vertical temperature gradient of 1 K/m, which appear to be reasonable values, produces a subsidence heating rate of 3.6 K/hr. Note that according to ERA‐Interim the average wintertime vertical velocity at 10 m above the surface amounts to approximately −0.6 mm/s) at Dome C (Appendix A).

**Figure 12:**
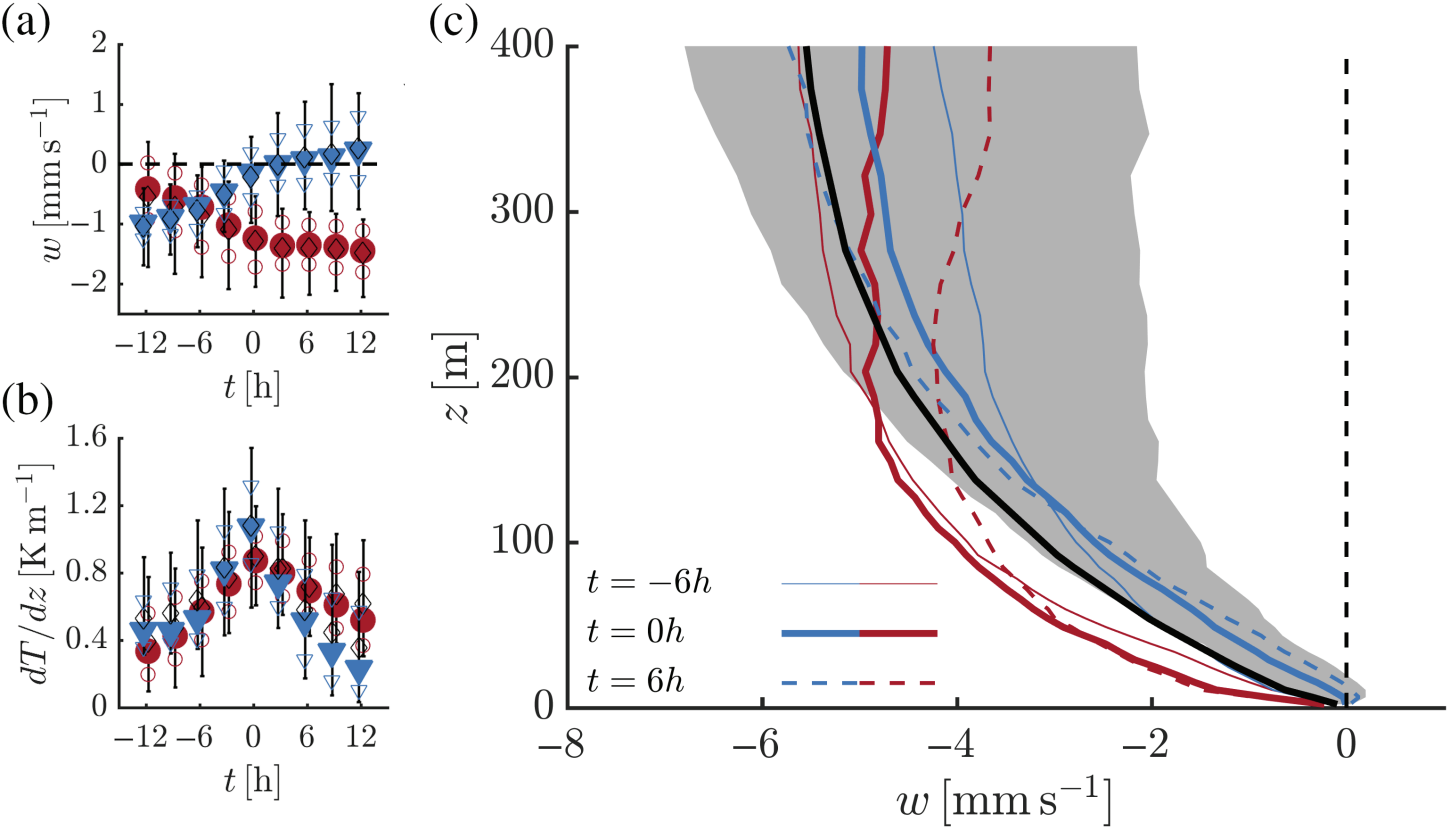
Composite time series of vertical velocity (a) and the vertical temperature gradient (b) at 10 m. Colours and symbols are as for Figure 5. Average profiles of the vertical velocity for inversion formation (red) and erosion (blue) at *t* = −6, 0 and 6 hr (c). The shaded area indicates the spread (25th and 75th percentiles) for the case of inversion erosion (*t* = 0 hr). The solid black line indicates the average wintertime profile [Colour figure can be viewed at wileyonlinelibrary.com].

Figure 12a shows composite time series of the vertical velocity in mm/s as diagnosed at 10 m height for both transition types. Clearly, the vertical velocities are not constant in time, indicating a change in the large‐scale conditions. This is not surprising, as earlier results showed that changes in the inversion strength are associated with variations in the geostrophic wind speed (Figure 7). The two transition types show essentially mirrored behaviours. According to Figure 12b, the vertical gradient of temperature maximizes around *t* = 0 hr at values of approximately 1 K/m.

To put the variation in the 10‐m vertical velocity into a broader perspective, Figure 12c presents vertical profiles at *t* = −6, 0 and 6 hr. Close to the ground the median values for both transitions types are clearly different, while at higher levels all profiles qualitatively resemble the average wintertime profile. The shaded area illustrates that large variability exists between the selected cases.

### 4.4 Fluctuations in the 40‐m inversion

We have demonstrated that changes in the depth of the turbulent layer as a result of a changing external forcing (geostrophic wind speed and subsidence) play a dominant role in the relation between the 10‐m inversion strength and the 10‐m wind speed. However, the choice of the 10‐m level as a reference is arguably arbitrary. Here, we discuss the impact of taking the 40‐m level as a reference. Based on the observations, we select all time intervals in which the temperature difference between 40 m and the surface increased or decreased continuously by more than 15 K (*LW*_*d*_ < 100 W/m^2^) in a similar way as done previously for the 10‐m inversion. In total, 74 (87) events were found for which the 40‐m inversion strength increased (decreased) accordingly.

**Figure 13:**
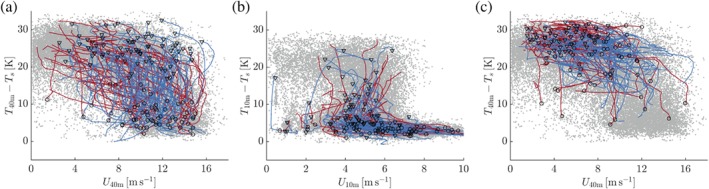
Relation between the 40‐m inversion strength versus the 40‐m wind speed (a,c) and the 10‐m inversion strength versus the 10‐m wind speed (b). Red (blue) lines indicate time series of cases with significant increase (decrease) (>15 K) in the 40‐m (a,b) and 10‐m (c) inversion strengths. Circles (triangles) indicate the starting point of each inversion formation (erosion) event [Colour figure can be viewed at wileyonlinelibrary.com].

Figure 13a presents the relation between the 40‐m inversion strength and the 40‐m wind speed. Qualitatively, the results resemble the relation between the 10‐m inversion and the 10‐m wind speed: two regimes appear to exist, with strong inversions for weak winds and relatively weak inversions for high wind speeds, separated by a narrow wind speed band in which the transition between the regimes takes place. At 40 m the transition occurs at a higher wind speed than at 10 m. Weak 40‐m inversions are relatively rare as they require strong mechanical forcing conditions that do not occur very often (cf. Figure 7).

Figure 13b shows the relation between the 10‐m inversion and the 10‐m wind speed as before, but now the time series indicate the selected cases of significant change in the 40‐m inversion. Clearly, when a transition occurs at 40 m, most of the time the 10‐m inversion is already in the weakly stable state. On the other hand, Figure 13c shows that when a transition occurs at 10 m, the 40‐m inversion is mostly in its very stable state.

We conclude that, qualitatively speaking, transitions in the 40‐m and 10‐m inversion strengths are manifestations of the same phenomenon. On the one hand, there is a state with very strong stratification in which the respective level is uncoupled from the surface. On the other hand, there is a state where turbulent mixing transports heat towards the surface leading to a much weaker inversion strength. At both levels, turbulence‐induced downward transport of momentum leads to a highly nonlinear relation between inversion strength and wind speed. The results presented here suggest that for an increasing geostrophic forcing the boundary layer deepens and progressively higher levels experience a transition from a very stable to a weakly stable state.

## 5 DISCUSSION

The results presented here show that in conditions typical for the Antarctic Plateau, the conceptual framework of van de Wiel *et al*. (2017) needs refinement. They stated that at a particular reference level a single “threshold” wind speed exists below which the boundary layer collapses as a whole. This rationale was based on an earlier study by van de Wiel *et al*. (2012a) who studied the collapse of the turbulence from a theoretical point of view by analysing a cooled channel flow. It was shown that the collapse of turbulence is essentially caused by the fact that the sustainable heat flux in stratified boundary layers is limited to a maximum under a given mechanical forcing. They proved that when the pressure gradient force falls below a certain threshold value this maximum sustainable heat flux can no longer compensate for the radiative loss and turbulence will become very weak. For application in real atmospheric boundary layers a so‐called velocity crossing level with constant wind speed in time was identified that served as a proxy for the external mechanical forcing. Following this rationale, Vignon *et al*. (2017b) adopted the 10‐m level as a reference for Dome C, as at this height the wind speed was found to be relatively constant in time (based on summertime data).

The concept of maximum sustainable heat flux in combination with the crossing level proved a good starting point for describing regime transitions of atmospheric boundary layers both at Cabauw, the Netherlands (van de Wiel *et al.,* 2012b; Monahan *et al.,* 2015; van Hooijdonk *et al.,* 2015) and at Dome C (Vignon *et al.,* 2017b). In particular, a sharp transition was found when plotting the strength of the steady‐state inversion at the crossing level versus the ambient wind speed (van de Wiel *et al*. 2017). Hence, with a priori knowledge of the wind, the corresponding inversion strength can be predicted.

When considering the *boundary layer as a whole* (without prior knowledge of the wind) the situation becomes more complex as increasing forcing leads to a deepening of the turbulent layer. For example, the present results indicate that at Dome C the regime transition at 10‐m height is related to the depth of the turbulent layer being thinner or thicker than 10 m. Thus, the 10‐m wind speed is an internal parameter (say “consequence”) of the system rather than a proper proxy of the external (mechanical) forcing as it is modified by turbulent stress divergence: as shown in Figure 8 the stress divergence at this level effectively counteracts the tendency in the geostrophic wind. This explains why van der Linden *et al*. (2017) and Baas *et al*. (2018) found much smoother regime transitions when taking the geostrophic wind speed rather than an observed boundary layer wind speed as the independent parameter.

In contrast to other locations such as Cabauw, at Dome C subsidence plays an important role in the formation of strong temperature inversions during the regime transition, as shown by previous results. It would therefore be interesting to extend the conceptual framework with this important external parameter.

Finally, some interesting positive feedback may potentially occur. A strengthening surface inversion over Dome C could lead to increased katabatic outflow around the dome (King *et al.,* 2001). This would lead to an increasingly divergent flow pattern, which would then act to increase the subsidence (van de Berg *et al.,* 2008). The present study suggests that increased subsidence promotes the formation of even stronger inversions, hence further strengthening the katabatic flow. Presumably, at some stage negative feedback will come into play in order to restore equilibrium. This reasoning illustrates the close relation between the boundary layer processes and the large‐scale circulation. Future research is needed to clarify to what extent feedback mechanisms such as those described above occur in reality.

## 6 CONCLUSIONS

The persistent surface‐based temperature inversion during the austral winter is one of the most prominent features of the climate of the Antarctic Plateau. The difference between the maximum tropospheric temperature and the surface temperature exceeds 25 K most of the time. The depth of the inversion layer consists of several hundreds of meters. Closer to the ground, previous studies indicated intriguing nonlinear behaviour between the inversion strength and the wind speed at a particular level. For instance, Vignon *et al*. (2017b) reported sudden transitions between strong (associated with weak winds) and weak near‐surface inversions (strong winds).

In this work, we studied the transition from weak to very strong near‐surface inversions and vice versa in detail by analysing 6 years of observations from a 45‐m measuring tower operated at the French–Italian Antarctic station Concordia at Dome C.

We selected all events for which the 10‐m inversion strength (*T*_10m_ – *T*_s_) increased or decreased continuously by at least 15 K. Events during which the incoming long‐wave radiation exceeded 100 W/m^2^ were excluded from the analysis. For both subsets, composite time series and vertical profiles of relevant variables were constructed. A similar analysis was performed on results from an atmospheric single‐column model (SCM). The SCM was driven by large‐scale forcings obtained from a regional climate model. Overall, the SCM results reproduced the observed characteristics of the transitions in the near‐surface inversion remarkably well.

Surprisingly, during the transitions, variations in the surface temperature were found to be much smaller than for the 10‐m temperatures. That is to say, erosion of strong inversions was predominantly the result of a decrease in *T*_10m_, while the formation of the 10‐m inversion was mainly the result of an increase in *T*_10m_. This contrasts strongly with conditions at midlatitudes, where the evolution of the near‐surface inversion strength is largely driven by changes in the surface temperature.

Model results indicate that the erosion and formation of the 10‐m inversion are mainly driven by changes in the geostrophic wind speed, *U*_*g*_, and by large‐scale subsidence. The interplay between changes in forcing conditions, the depth of the turbulent layer and the divergence of turbulent fluxes explains the nonlinearity in the relation between the inversion strength and the wind speed at a particular level. For instance, in the case of weak geostrophic forcing, the depth of the turbulent layer is generally less than 10 m and the 10‐m inversion strength may be 25 K. When in this case *U*_*g*_ accelerates, both the 10‐m wind speed and the depth of the turbulent layer will increase. As the depth of the turbulent layer exceeds the 10‐m level, stress divergence starts counteracting the increase in *U*_10m_. At the same time, strong heat flux divergence reduces *T*_10m_. The end result is a weak 10‐m inversion in strong wind conditions.

Subsidence heating plays a crucial role in the formation of the near‐surface inversion. Even at 10 m above ground level, heating rates of almost 4 K/hr are reached. Although the importance of subsidence heating over the dome‐shaped Antarctic Plateau is widely acknowledged, this still comes as a surprising result. One of the factors leading to this large value is the very strong vertical temperature gradient close to the surface.

The present study provides a detailed analysis of regime transitions in the surface‐based temperature inversion in the first tens of meters above the Antarctic Plateau in the polar winter. Again, the unique long‐term observational dataset of Dome C proved to be a valuable natural laboratory for atmospheric boundary layer studies. Utilizing the good correspondence of the mean vertical profiles of the atmospheric state, results from a numerical atmospheric model could be used to identify and unravel the dominant processes of this intriguing phenomenon.

## Figures and Tables

**Figure A1:**
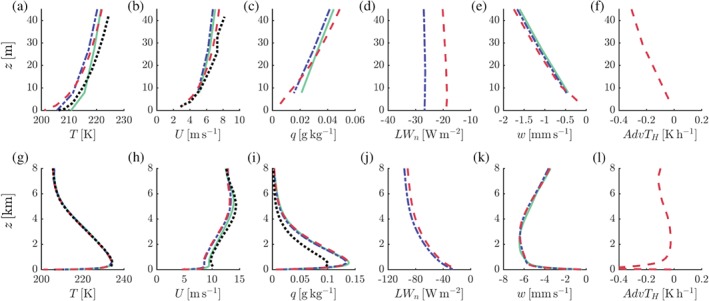
Averaged wintertime vertical profiles of temperature (a,g), wind speed (b,h), specific humidity (c,i), net long‐wave radiation (d,j), vertical velocity (e,k) and horizontal temperature advection (f,l). Observations are indicated in black (dotted lines), the SCM in red (dashed lines), the RACMO driver model in blue (dashed‐dotted lines), and ERA‐Interim in green (solid lines). Top panels indicate the lowest 45 m (with tower observations), bottom panels the lowest 8 km (with radio soundings) [Colour figure can be viewed at wileyonlinelibrary.com].

**Figure B1:**
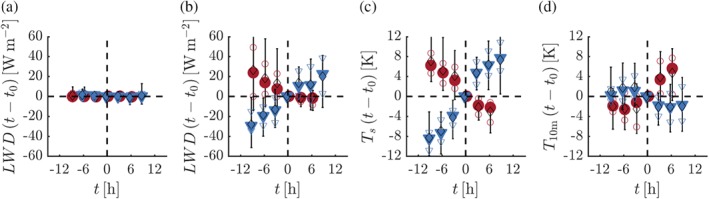
Observed composite time series of the change in *LW*_*d*_ (a,b), *T*_s_ (c) and *T*_10m_ (d) for selected transition events. Events that satisfy (a) and exceed (b–d) the imposed threshold value on *LW*_*d*_ are shown. Events of inversion formation are indicated in red (circles) and events of inversion erosion in blue (triangles). Filled symbols indicate median values, open symbols the 25th and 75th percentiles, and bars the 10th and 90th percentiles. Open black diamonds indicate average values. Only data points for which more than 25% of the selected events are available are indicated [Colour figure can be viewed at wileyonlinelibrary.com].

## APPENDIX A 1 BASIC CLIMATOLOGY OF THE WINTERTIME TROPOSPHERE AT DOME C

This study uses results from the single‐column model (SCM) of the Regional Atmospheric Climate Model (RACMO). The SCM is driven by a three‐dimensional (3D) climate simulation in evaluation mode of a modified version of RACMO that was specifically adapted to polar conditions. On its turn, the 3D RACMO run was forced by ERA‐Interim. Here a basic comparison of averaged modelled and observed vertical profiles is presented. At the same time, the large‐scale “synoptic” background setting is discussed.

Figure A1 shows vertical profiles for the SCM, RACMO (3D) and ERA‐Interim. Observations are added where available. In the troposphere, the differences between the models are small. Modelled wind and temperature profiles agree very well with profiles from radio soundings. In the lowest 50 m, vertical gradients of temperature and wind speed are better captured by the SCM than by the 3D models that each apply too much vertical mixing. The SCM show a cold bias of several degrees. Consequently, the net long‐wave fluxes are smaller than in the 3D model. Differences in vertical radiative flux divergence are small.

The troposphere over the Antarctic Plateau is characterized by persistent large‐scale descending motions. The largest values are attained at about 500 m above ground level, which roughly corresponds to the top of the surface‐based inversion. Even closer to the surface, the predominantly southerly flow advects cold air from the interior of the continent. The combination of large‐scale descending motions and horizontal advection of colder air in the boundary layer is characteristic of the dome‐shaped interior of the Antarctic continent (James, 1989; King and Turner, 1997; van de Berg *et al.,* 2008).

## APPENDIX B 1 REGIME TRANSITIONS AND SYNOPTIC WARMING EVENTS

At Dome C, synoptic warming events are associated with inland advection of relatively moist and warm air from coastal regions. As a result, they are characterized by high values of *LW*_*d*_. Our analysis only includes data records with *LW*_*d*_ < 100 W/m^2^. For the sake of argument, we repeated the selection of transition events with a significant change of the 10‐m inversion without the limiting threshold on *LW*_*d*_. As a result, 27 (58) *additional* cases were found with significant formation (erosion) of the 10‐m inversion strength.

Figure B1 shows that these additional events display very different characteristics compared to the reference transition events that satisfy the 100 W/m^2^ upper limit on *LW*_*d*_. The additional transition cases show a strong decrease (increase) of *LW*_*d*_ in the case of inversion formation (erosion). Changes in *LW*_*d*_ amount to 60 W/m^2^, while for the reference cases *LW*_*d*_ is approximately constant in time. This difference in the radiation budget has major implications for the dynamics of the temperature time series. For the transition cases that exceed the threshold on *LW*_*d*_, the 10‐m inversion strength changes mainly as a result of variations in *T*_s_ (Figure B1c). In contrast, for the reference set of transition events it is the 10 m temperature that shows the largest variation (Figure 5a,b). Furthermore, when the *LW*_*d*_ threshold is applied, in almost all events the flow is continuously from southerly directions (i.e., continental air). Events that exceed the *LW*_*d*_ threshold are associated with more easterly and northerly directions characteristic of warming events (not shown).

We conclude that the transition events that exceed the threshold on *LW*_*d*_ share many characteristics of the large‐scale warming events (cf. Gallée and Gorodetskaya, 2010). However, by including only data records with *LW*_*d*_ < 100 W/m^2^ the impact of warming events is largely avoided.
